# Autosomal recessive *VWA1*-related disorder: comprehensive analysis of phenotypic variability and genetic mutations

**DOI:** 10.1093/braincomms/fcae377

**Published:** 2024-10-28

**Authors:** Sara Nagy, Alistair T Pagnamenta, Elisa Cali, Hilde M H Braakman, Juerd Wijntjes, Benno Kusters, Marc Gotkine, Orly Elpeleg, Vardiella Meiner, Jerica Lenberg, Kristen Wigby, Jennifer Friedman, Luke D Perry, Alexander M Rossor, Anna Uhrova Meszarosova, Dana Thomasova, Saiju Jacob, Mary O'Driscoll, Lenika De Simone, Dorothy K Grange, Richard Sommerville, Zahra Firoozfar, Shahryar Alavi, Mahta Mazaheri, Jevin M Parmar, Phillipa J Lamont, Veronica Pini, Anna Sarkozy, Francesco Muntoni, Gianina Ravenscroft, Eppie Jones, Declan O'Rourke, Melissa Nel, Jeannine M Heckmann, Michelle Kvalsund, Musambo M Kapapa, Somwe Wa Somwe, David R Bearden, Arman Çakar, Anne-Marie Childs, Rita Horvath, Mary M Reilly, Henry Houlden, Reza Maroofian

**Affiliations:** Centre for Neuromuscular Diseases, Department of Neuromuscular Diseases, UCL Queen Square Institute of Neurology, London WC1N 3BG, UK; Department of Neurology, University Hospital Basel, University of Basel, Basel 4031, Switzerland; NIHR Oxford Biomedical Research Centre, Centre for Human Genetics, University of Oxford, Oxford OX3 9DU, UK; Centre for Neuromuscular Diseases, Department of Neuromuscular Diseases, UCL Queen Square Institute of Neurology, London WC1N 3BG, UK; Department of Pediatric Neurology, Amalia Children’s Hospital, Radboud University Medical Center & Donders Institute for Brain, Cognition and Behavior, Nijmegen 6525 GA, The Netherlands; Department of Neurology and Clinical Neurophysiology, Donders Institute for Brain, Cognition and Behavior, Radboud University Medical Center, Nijmegen 6525 GD, The Netherlands; Department of Pathology, Radboudumc, Nijmegen 6525 GA, The Netherlands; Department of Neurology, Hadassah Medical Organization and Faculty of Medicine, Hebrew University of Jerusalem, Jerusalem 9112001, Israel; Department of Genetics, Hadassah Medical Center, Hebrew University Medical Center, Jerusalem 9574869, Israel; Department of Genetics, Hadassah Medical Center, Hebrew University Medical Center, Jerusalem 9574869, Israel; Rady Children’s Institute for Genomic Medicine, San Diego, CA 92123, USA; Rady Children’s Institute for Genomic Medicine, San Diego, CA 92123, USA; Rady Children’s Hospital, San Diego, CA 92123, USA; Department of Pediatrics, University of California San Diego, La Jolla, CA 92093, USA; Department of Pediatrics, University of California San Diego, La Jolla, CA 92093, USA; The Dubowitz Neuromuscular Centre, UCL Great Ormond Street, Great Ormond Street Hospital, London WC1N 1EH, UK; NIHR Great Ormond Street Hospital Biomedical Research Centre, UCL Great Ormond Street Institute of Child Health, London WC1N 1EH, UK; MRC International Centre for Genomic Medicine in Neuromuscular Diseases, London WC1N 3BG, UK; Centre for Neuromuscular Diseases, Department of Neuromuscular Diseases, UCL Queen Square Institute of Neurology, London WC1N 3BG, UK; Neurogenetic Laboratory, Department of Paediatric Neurology, and Institute of Biology and Medical Genetics, Second Faculty of Medicine, Charles University and University Hospital Motol, Prague 150 06, Czech Republic; Neurogenetic Laboratory, Department of Paediatric Neurology, and Institute of Biology and Medical Genetics, Second Faculty of Medicine, Charles University and University Hospital Motol, Prague 150 06, Czech Republic; Department of Neurology, University Hospitals Birmingham, Birmingham B15 2TT, UK; Institute of Immunology and Immunotherapy, University of Birmingham, Birmingham B15 2TT, UK; West Midlands Regional Clinical Genetics Service and Birmingham Health Partners, Birmingham Women’s and Children’s Hospital NHS Foundation Trust, Birmingham B15 2TG, UK; Division of Genetics, Genomics, and Metabolism, Ann & Robert H. Lurie Children’s Hospital of Chicago, Chicago, IL 60611, USA; Division of Neurology, Ann & Robert H. Lurie Children’s Hospital of Chicago, Chicago, IL 60611, USA; Department of Neurology at Washington University, Washington University School of Medicine, St. Louis Children’s Hospital, St. Louis, MO 63108, USA; Department of Neurology at Washington University, Washington University School of Medicine, St. Louis Children’s Hospital, St. Louis, MO 63108, USA; Palindrome, Isfahan 83714, Iran; Palindrome, Isfahan 83714, Iran; Department of Medical Genetics, School of Medicine, Shahid Sadoughi University of Medical Sciences, Yazd 97514, Iran; Dr. Mazaheri’s Medical Genetics Lab, Yazd 97514, Iran; Rare Disease Genetics and Functional Genomics Group, Harry Perkins Institute of Medical Research, Nedlands, WA 6009, Australia; Centre for Medical Research, University of Western Australia, Nedlands, WA 6009, Australia; Royal Perth Hospital, Perth, WA 6000, Australia; The Dubowitz Neuromuscular Centre, UCL Great Ormond Street, Great Ormond Street Hospital, London WC1N 1EH, UK; The Dubowitz Neuromuscular Centre, UCL Great Ormond Street, Great Ormond Street Hospital, London WC1N 1EH, UK; MRC International Centre for Genomic Medicine in Neuromuscular Diseases, London WC1N 3BG, UK; The Dubowitz Neuromuscular Centre, UCL Great Ormond Street, Great Ormond Street Hospital, London WC1N 1EH, UK; NIHR Great Ormond Street Hospital Biomedical Research Centre, UCL Great Ormond Street Institute of Child Health, London WC1N 1EH, UK; Rare Disease Genetics and Functional Genomics Group, Harry Perkins Institute of Medical Research, Nedlands, WA 6009, Australia; Centre for Medical Research, University of Western Australia, Nedlands, WA 6009, Australia; Genomics Medicine Ireland, Dublin D18 K7W4, Ireland; Children’s Health Ireland at Temple Street, Dublin, Dublin D01 XD99, Ireland; Neurogenomics Lab, Neuroscience Institute, University of Cape Town, Cape Town 7935, South Africa; Department of Medicine, Faculty of Health Sciences, University of Cape Town, Cape Town 7935, South Africa; Neurology Research Group, Neuroscience Institute, University of Cape Town, Cape Town 7935, South Africa; Department of Neurology, University of Rochester Medical Center, Rochester, NY 14618, USA; Department of Internal Medicine, University of Zambia School of Medicine, Ridgeway, Lusaka, Zambia; Department of Physiotherapy, University of Zambia School of Health Sciences, Lusaka, Zambia; Department of Paediatrics and Child Health, School of Medicine and Health Sciences, University of Lusaka, Lusaka, Zambia; Department of Neurology, University of Rochester Medical Center, Rochester, NY 14618, USA; Department of Educational Psychology, University of Zambia, Lusaka, Zambia; Centre for Neuromuscular Diseases, Department of Neuromuscular Diseases, UCL Queen Square Institute of Neurology, London WC1N 3BG, UK; Neuromuscular Unit, Istanbul Faculty of Medicine, Istanbul University, Istanbul 34093, Turkey; Department of Paediatric Neurology, Leeds Teaching Hospitals NHS Trust, Leeds LS1 3EX, UK; Department of Clinical Neurosciences, University of Cambridge, Cambridge CB2 2PY, UK; Centre for Neuromuscular Diseases, Department of Neuromuscular Diseases, UCL Queen Square Institute of Neurology, London WC1N 3BG, UK; Centre for Neuromuscular Diseases, Department of Neuromuscular Diseases, UCL Queen Square Institute of Neurology, London WC1N 3BG, UK; Centre for Neuromuscular Diseases, Department of Neuromuscular Diseases, UCL Queen Square Institute of Neurology, London WC1N 3BG, UK

**Keywords:** VWA1, neuromuscular disorders, neuromyopathy, recessive disorders

## Abstract

A newly identified subtype of hereditary axonal motor neuropathy, characterized by early proximal limb involvement, has been discovered in a cohort of 34 individuals with biallelic variants in von Willebrand factor A domain-containing 1 (*VWA1*). This study further delineates the disease characteristics in a cohort of 20 individuals diagnosed through genome or exome sequencing, incorporating neurophysiological, laboratory and imaging data, along with data from previously reported cases across three different studies. Newly reported clinical features include hypermobility/hyperlaxity, axial weakness, dysmorphic signs, asymmetric presentation, dystonic features and, notably, upper motor neuron signs. Foot drop, foot deformities and distal leg weakness followed by early proximal leg weakness are confirmed to be initial manifestations. Additionally, this study identified 11 novel *VWA1* variants, reaffirming the 10 bp insertion-induced p.Gly25ArgfsTer74 as the most prevalent disease-causing allele, with a carrier frequency of ∼1 in 441 in the UK and Western European population. Importantly, VWA1-related pathology may mimic various neuromuscular conditions, advocating for its inclusion in diverse gene panels spanning hereditary neuropathies to muscular dystrophies. The study highlights the potential of lower quality control filters in exome analysis to enhance diagnostic yield of VWA1 disease that may account for up to 1% of unexplained hereditary neuropathies.

## Teaser

Nagy *et al*. contributed to the better understanding of a recently described neuromuscular condition characterized by motor neuropathy and myopathic features due to recessive *VWA1* variants. They introduced new clinical features as well as new variants in the so far biggest patient cohort including European, Middle Eastern and African families.

## Graphical Abstract

**Graphical Abstract:**
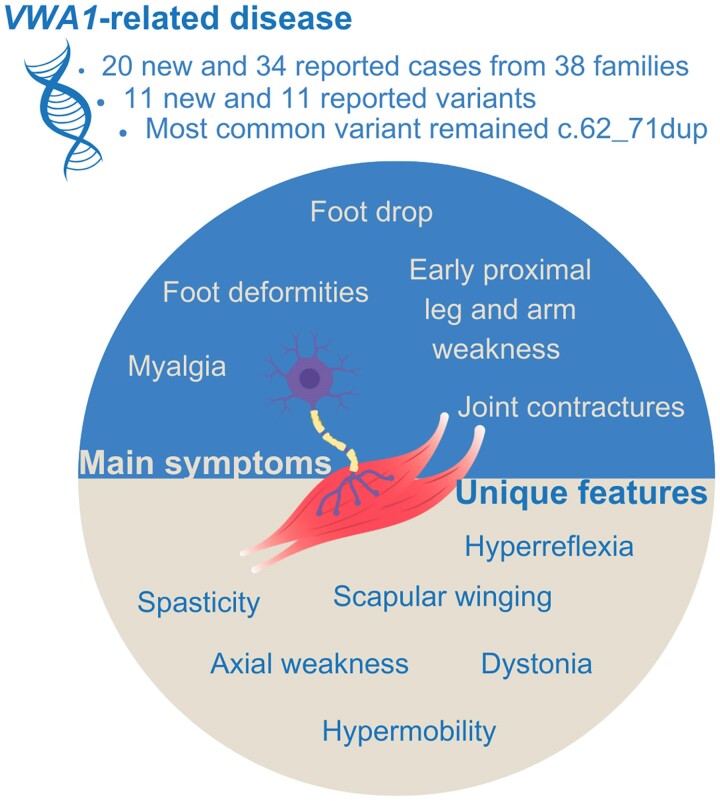

## Introduction

In early 2021, we reported on 17 individuals from 15 families exhibiting motor neuropathy associated with rare biallelic variants in von Willebrand factor A domain-containing 1 (*VWA1*).^1^ Among these, 10 families were part of the 100,000 Genomes Project, a substantial UK initiative demonstrating the feasibility of routine genome sequencing within the NHS.^2^ Notably, a relatively common Western European founder variant [NM_022834.5:c.62_71dup, p.(Gly25ArgfsTer74)] was identified in 14 out of 15 families, residing on a shared haplotype with an ancient origin. Parallel to our study, another cohort, assembled by Deschauer *et al.*,^3^ included 15 individuals from 6 families with biallelic *VWA1* variants.

Patients were characterized by a childhood or adult onset of a slowly progressive, non–length-dependent axonal hereditary motor neuropathy. Notably, there was significant involvement of the proximal lower limbs, surpassing that of the upper limbs in most cases. Histological and neurophysiological findings reported in the study by Deschauer *et al.* pointed towards both neuropathic and myopathic changes, leading them to define the condition as neuromyopathy. Nevertheless, the true myopathic nature of the disease remained controversial, with some proposing a primarily neurogenic pathology and a secondary myopathic process.

To comprehensively characterize the clinical spectrum of this highly variable neurological condition, we established a new cohort comprising 20 patients from 15 independent, mostly non-consanguineous families. Thorough clinical and paraclinical assessments, including nerve conduction studies, muscle ultrasound and biopsy data, were collected. Our findings highlight the diverse age of onset and phenotypic presentations of the disease, which can mimic various hereditary neuromuscular conditions, ranging from Charcot–Marie–Tooth disease to spinal muscular atrophy (SMA), hereditary spastic paraplegia spectrum and muscular dystrophies. We report previously undocumented variants and reaffirm, in line with initial reports from 2021, that c.62_71dup stands out as the most prevalent pathogenic variant.

## Materials and methods

### Family identification and clinical and genetic investigation

We identified 15 new families with biallelic *VWA1* variants through the GeneMatcher platform,^4^ supported by a collaborative network of international researchers and screening various genetic databases. Exome analysis was employed for 10 families, while genome sequencing was conducted for the remaining 5. We gathered comprehensive clinical details, neurophysiological data, muscle imaging, muscle biopsy specimens, brain imaging and visual materials such as photos and videos of affected individuals. Informed consent and authorization for publishing photos and videos (if applicable) were obtained from each patient or their legal representatives.

Procedures were reviewed and approved by the appropriate institutional review committee.

## Results

### Clinical characterization

**Figure 1:**
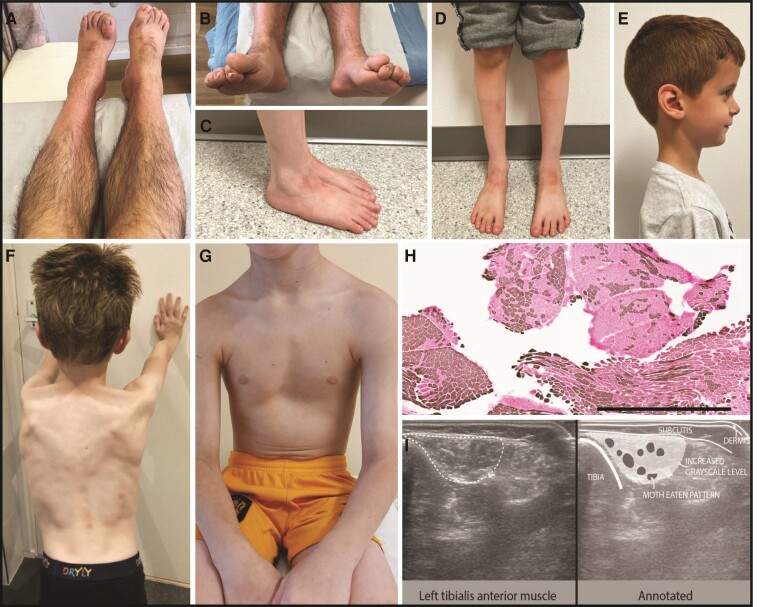
**Clinical and imaging features of affected individuals with VWA1 disease.** Images of affected individuals from Patients 1 (**A and B**), 3 (**C–E**), 8 (**F**) and 13 (**G**) showing moderate lower distal limb atrophy (**A and D**) with pes planus (**B and C**), overlying second toe (**B**), upgoing toes (**B**), dolichocephaly and frontal bossing (**E**), scapula alata (**F**) and pes excavatum (**G**). (**H**) Muscle biopsy of the vastus lateralis muscle of Patient 8 shows extensive neurogenic abnormalities with many areas of fibre type grouping seen by ATPase (pH 4.6) staining. Light brown fibres correspond to Type 2B or 2C, while dark brown-stained fibres are Type 1 and red fibres Type 2A. Scale bar: 1 mm. (**I**) Muscle ultrasound of the left tibialis anterior from the same patient shows a moth-eaten pattern consisting of round, dark areas with remaining viable motor units, surrounded by tissue with increased greyscale level reflecting permanently denervated and fibrosed muscle tissue.

In our cohort of deeply phenotyped individuals (*n* = 20, aged 2–74 years) (Fig. 1A–G; Supplementary Table 1 and Videos 1–4), 12 exhibited solely European ancestries, while others were coming from diverse backgrounds including mixed British–Pakistani, European–Arab, Mexican, Moroccan Jewish, Iranian and African families. Consanguinity was reported in two families of Iranian and Pakistani origin. The age of onset varied from birth to 12 years, with consistent early involvement of the distal lower limbs, presenting as various foot deformities, gait disturbances, frequent falls and ankle contractures. Notably, all cases except two exhibited distal lower limb weakness with mostly foot drop. One individual showed asymmetric involvement with one-sided foot drop, pes cavus on the left and pes planus on the right (Patient 15, Family 13). Nine individuals also manifested proximal lower limb or axial weakness, and six developed weakness in the upper limbs within the first three decades of life. One child showed only proximal lower limb weakness, while her younger brother retained motor strength but exhibited dystonic features in his legs (Patients 19 and 20 from Family 15). The most pronounced weakness was observed in the distal lower limbs, with muscle strength ranging from Grades 1 to 3 for toe and foot dorsiflexion or plantarflexion. In contrast, muscle strength in the proximal lower limbs, as well as in the distal and proximal upper limbs, was at least Grade 4, except for Patient 12 (Family 11) who exhibited Grade 3–4 weakness in the proximal legs and Grade 2–3 weakness in the hands and for Patient 20 (Family 15) who had Grade 3 weakness in the hip abductors. Mild muscle atrophy, predominantly affecting the distal legs, was common. Additional clinical signs included skeletal deformities (*n* = 5), spasticity (*n* = 2), upgoing plantars (*n* = 2), scapular winging (*n* = 2), tongue fasciculations (*n* = 2), multifocal dysmorphic signs including dolichocephaly, frontal bossing, high arched palate, bilateral fifth finger clinodactyly and partial 2,3 syndactyly in feet (*n* = 1), hypermobility/hyperlaxity (*n* = 1) and dystonia (*n* = 1). No associated neurocognitive symptoms were observed. Cardiac or respiratory involvement was not reported. Despite the diverse clinical manifestations, all affected individuals exhibited a slow disease progression and remained ambulatory at the time of investigations.

### Diagnostic findings

Blood tests unveiled elevated creatine kinase (CK) levels in the majority of cases, with the highest value reaching up to 2400 U/L. Nerve conduction studies consistently indicated axonal motor neuropathy with sensory involvement in three cases, accompanied by chronic neuropathic changes in needle EMG. Myopathic EMG pattern was described in only one individual. Histological data were available for three cases, revealing a dystrophic pattern in one with multiple ring fibres, while the other two exhibited extensive fibre grouping indicative of neurogenic changes (Fig. 1H). None of these myopathological data were available for review, and only one patient agreed to publish the neuropathological images. In Families 8 and 12, muscle ultrasound demonstrated a moth-eaten pattern in the lower leg muscles, which can be seen in long-standing denervation without reinnervation.^5^ Strongly enhanced ultrasound intensity was seen in the rectus femoris, vastus lateralis and tibialis anterior muscles of the leg as well as in the deltoid, biceps and flexor digitorum profundus muscles of the arms while nerve ultrasound remained normal in all three cases investigated (Fig. 1I). Brain MRI was conducted in eight individuals, uncovering non-specific minor changes with patchy white matter abnormalities in three of them, most prominently in the frontal regions.

### Genetic analysis

**Figure 2:**
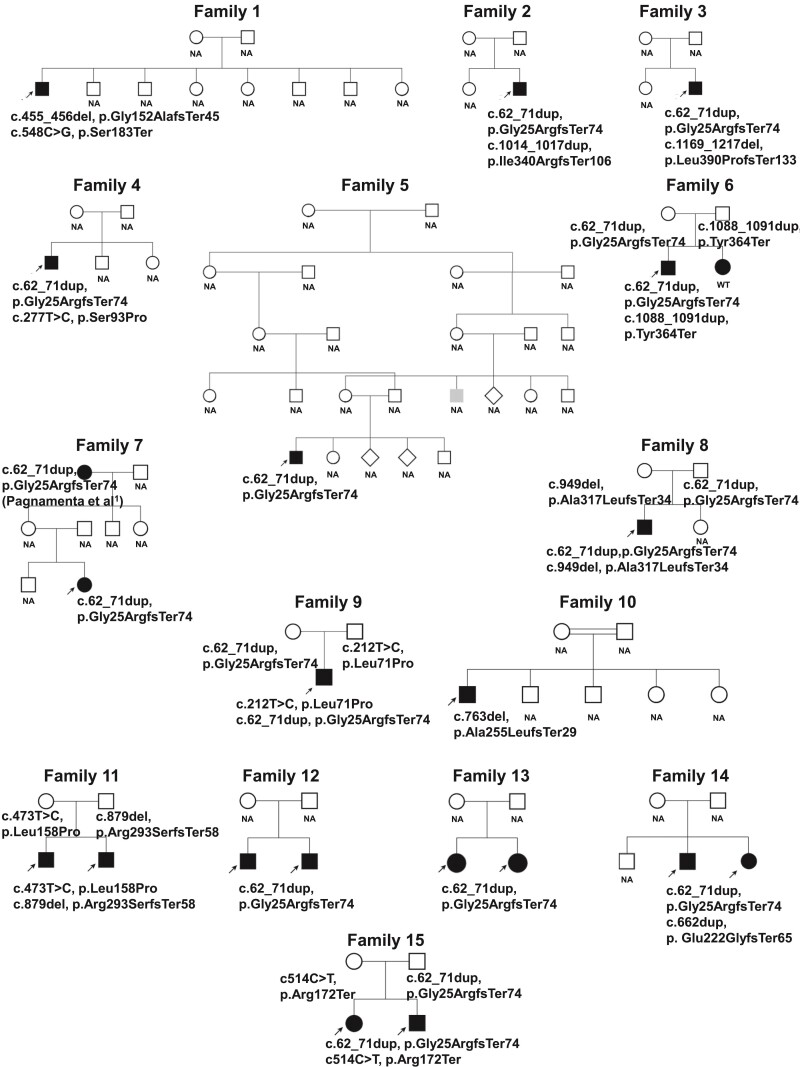
**Pedigrees of 15 families described in the study.** Filled symbols with arrow indicate phenotypic features consistent with VWA1-related disease. Grey shading in Family 5 indicates an individual with walking problems. In Family 6, sibling of the affected patient showed a similar phenotype but had no disease-causing variants in *VWA1*. Note that the grandmother of the affected individual in Family 7 corresponds to the described case in Family 7 in Pagnamenta *et al.*^1^ NA, not available; UK, unknown; WT, wild type.

With the exception of Patient 7 (Family 7), who is the grandmother of F7 described previously,^1^ none of these new cases are known to be directly related to families from our previously described cohort (Fig. 2). Consistent with our earlier findings, c.62_71dup was again the predominant disease-associated allele, observed in 12/15 families. In four families, this recurrent 10 bp duplication was detected in the homozygous state, and in two cases, we were able to review genomic data to assess the five SNPs that define the European founder haplotype. Family 13 (recruited in Ireland) had the proband homozygous for the non-reference alleles at rs76947392, rs188670510 and rs144707149 but heterozygous for rs114330234 and rs78379068. These data support our earlier study, confirming the presence of a founder haplotype. This result also helped infer homozygosity for the 10 bp duplication, which was helpful given the repetitive nature of the variant. This was later confirmed by Sanger sequencing. Heterozygosity for the two distal most SNPs implied a recombination event that had occurred in the maternal lineage. In contrast, Patient 5 (Family 5) was of Iranian ethnicity and, although homozygous for c.62_71dup, did not appear to have the European founder haplotype. The African ancestry Patients 17 and 18 were heterozygous for the common c.62_71dup and a novel likely pathogenic frameshift variant in Exon 3 (c.662dup).

As the 10 bp duplication is found at a locus where there is already a 2 × 10 bp repeat in the reference (2×GGCGCGGAGC), variant recurrence on a different haplotype is not unexpected. The maximum population allele frequency of the most common variant across all databases remained the highest in the European non-Finnish with 0.093% in gnomAD v4.0.0 and 0.118% in UK Biobank (https://afb.ukbiobank.ac.uk, accessed 28 March 2024) (Supplementary Table 2).

**Figure 3:**
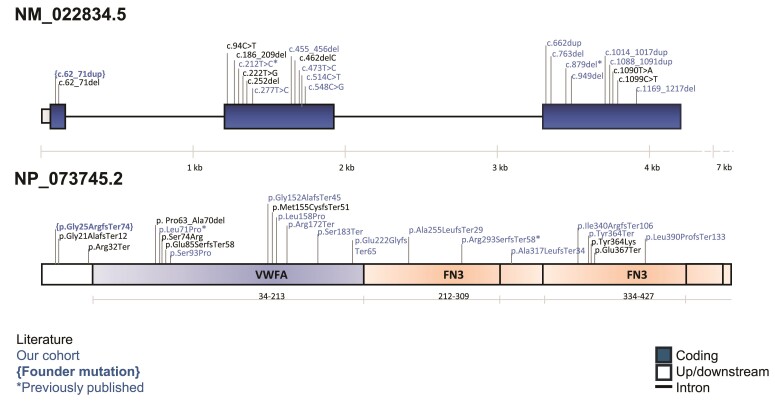
**Genomic position of *VWA1* variants.** Of the 13 *VWA1* variants (NM_022834.5 and NP_073745.2 correspond to *VWA1* mRNA and protein reference sequence accession numbers, respectively) described in the study, only the founder variants p.Gly25ArgfsTer74, p.Leu71Pro and p.Arg293SerfsTer58 have been described in the literature to be associated with disease.^1-3^

Of the other 13 variants identified, 3 were missense changes involving proline residues with supportive *in silico* scores (e.g. CADD = 24.9/27.6). The other 10 were all predicted to result in loss of function (3 stop-gains and 7 frameshifts) (Fig. 3; Supplementary Table 3). Of the 13 variants, 12 were seen in the compound heterozygous state, and 8 were in trans with c.62_71dup. The PM3 criteria from the ACMG variant interpretation framework^6^ can therefore be used to support pathogenicity. Of these 13 additional variants, we note that 5 are listed in ClinVar and 5 are present in dbSNP (rs750227698, rs747475277, rs1638567522, rs746212067 and rs749945726). The maximum population allele frequency seen across the set of additional variants was for p.(Leu71Pro) (Family 9), which was at an allele frequency of 50/981082 in the UK Biobank. We note that the variant was also identified in Case 2 described by Gable *et al.*,^7^ while the variant c.879del, p.(Arg293SerfsTer58), was reported previously by Deschauer *et al*.^3^

Patient 3 with dysmorphic signs also received a microarray analysis without any pathogenic results. Given the broad phenotypic presentation of the condition, several other patients received preceding genetic investigations including neuropathy/neuromuscular disorder panel, movement disorder/dystonia panel, testing for Duchenne muscle dystrophy, facioscapulohumeral muscular dystrophy and SMA.

## Discussion

The recent identification of truncating biallelic variants in *VWA1* has unveiled a novel hereditary neurological condition with a phenotype that continues to expand, encompassing non–length-dependent axonal motor neuropathy with early involvement of proximal limbs and upper extremities. Gene discovery faced delays in the majority of the 34 reported cases, attributed in part to challenges related to the high GC content and subsequent low coverage of the 10 bp repeat expansion in individuals with the most common recurrent mutation.^1^

From a clinical standpoint, the condition may go unnoticed for years to decades due to its relatively benign nature, often presenting mild neurological deficits, the absence of prominent muscle atrophy and slow disease progression. Nonetheless, early-onset foot deformities, contractures, and gait abnormalities have been consistently reported in nearly all individuals with VWA1-related disorder, suggesting an early or even infantile disease onset rather than adult onset.

**Figure 4:**
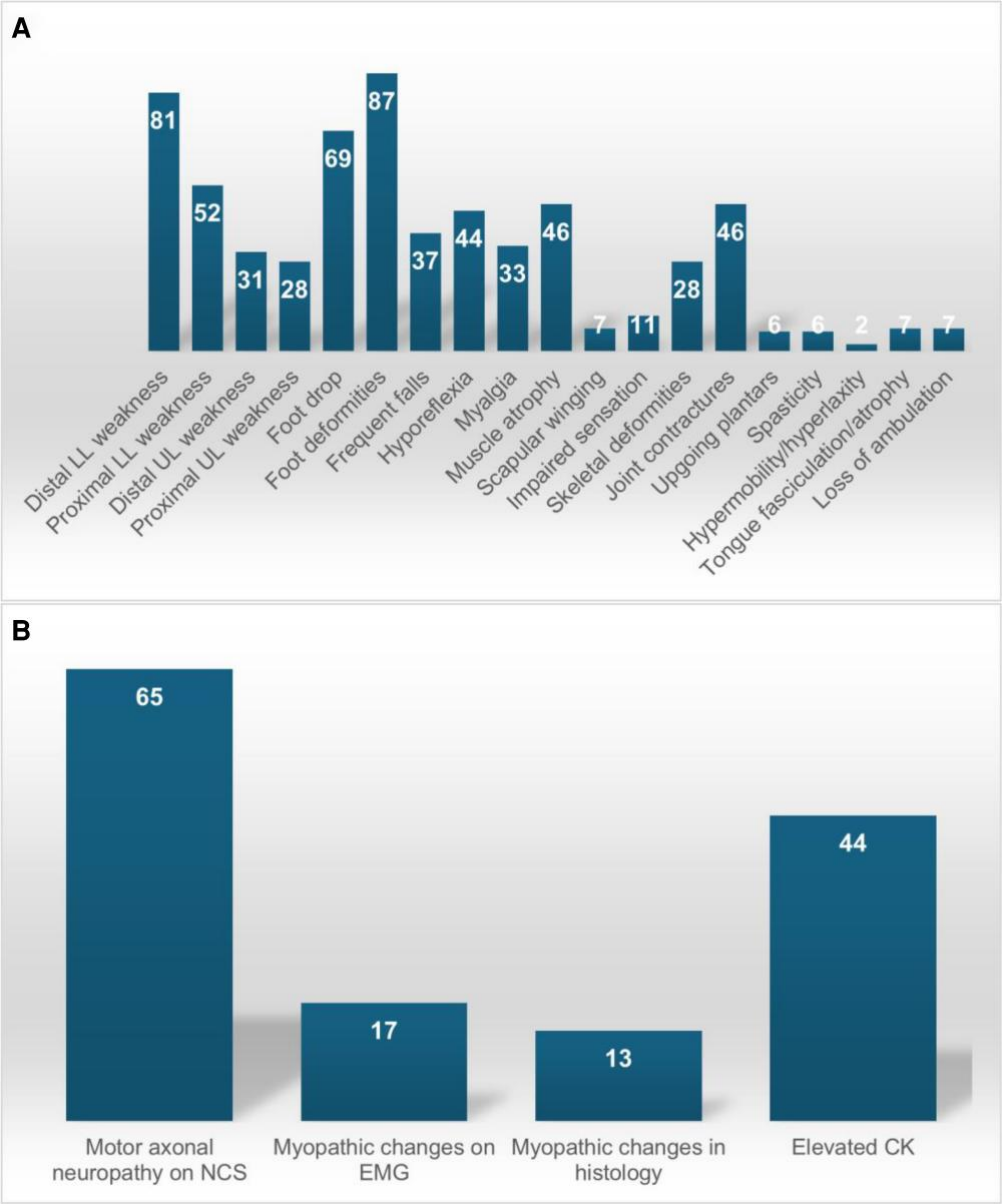
**Major features in individuals with VWA1 disease.** (**A**) The frequency of selected clinical symptoms. (**B**) The frequency of diagnostic signs in percentage (%). Results are based on data from 54 patients (*n* = 20 from presented cohort and *n* = 34 from previous publications).^1,2,7^ CK, creatine kinase; EMG, electromyography; LL, lower limb; NCS, nerve conduction studies; UL, upper limb.

While the most common features of VWA1-related disease include foot drop, foot deformities and distal lower limb weakness, about half of the so far reported individuals develop proximal leg weakness over time, and approximately one-third experience upper limb involvement (Fig. 4). The condition typically presents isolated motor neuropathy with primarily axonal involvement and chronic neurogenic changes as the typical neurophysiological finding. However, individuals in this cohort demonstrated additional features such as frequent falls, hyperreflexia and asymmetric presentation. Unique features of VWA1 disease include scapular winging, tongue fasciculations and, recently reported,^7^ pyramidal signs such as upgoing plantars and/or spasticity in the lower limbs. Additionally, this cohort identified previously undescribed features such as hypermobility and hyperlaxity, axial weakness including weakness of the trunk and neck flexors, dystonic features and dysmorphic signs. Importantly, none of the current cases experienced loss of ambulation compared with the 12% reported previously.^1,3,7^

Many clinical features of VWA1-related disorder are non-specific, meaning that it can potentially be mistaken for a range of hereditary conditions, particularly when the phenotype is not fully expressed. Early stages of the disease may resemble hereditary (sensory) motor neuropathies, and proximal leg weakness can mimic lower limb–predominant SMA or TGF-related hereditary neuropathy with proximal involvement.^8-10^ Hyperreflexia, especially when combined with spasticity, might mimic conditions on the hereditary spastic paraplegia spectrum. Rare features such as scapular winging in association with motor neuropathy can also be seen in patients with *TRPV4*, or *SORD* variants, and in those carrying *DYNC1H1*, and *BICD2* variants, where early proximal lower limb involvement is common.^11-13^ Tongue fasciculation and/or atrophy favours the diagnosis of juvenile amyotrophic lateral sclerosis associated with several genes including *SETX*, *SPTLC1*, *SPTLC2*, *ALS2*, *FUS* and *SIGMAR1*. Further, this symptom has been reported in *TRPV4* variant carriers.^13^ GARS disease is characterized by distal weakness of predominantly the upper limbs, but an associated phenotypic presentation more on the SMA spectrum with severe distal and proximal weakness can rarely present with tongue fasciculations.^13^ The combination of proximal leg weakness, myalgia and elevated CK levels suggested a potentially combined neuro-myopathological origin of VWA1-related disorder, aligning with the concept of neuromyopathy introduced in previous studies.^12,14^ Hereby, needle EMG was suggestive for myopathic changes in some previously reported cases, while muscle MRI showed fatty degeneration predominantly of the vastus lateralis and the anterior compartment of the lower leg, with myopathic pattern in single cases.^1,2^ Limited number of patients received muscle biopsy with some showing histological signs of myopathy.^1,2^ In our cohort, two patients had CK levels > 1500 U/L, with one of them exhibiting myopathic changes on muscle biopsy and electrodiagnostic. However, paraclinical testing indicated primarily neurogenic changes in all other cases. Muscle MRI was not performed in our cohort; however, three patients received detailed muscle ultrasound showing increased intensity in selected muscles of predominantly the upper arm and the anterior leg, with ‘moth-eaten’ pattern compatible with chronic denervation without reinnervation seen in one patient.

A recent proteomic study revealed increased immunostaining for C-reactive protein in the extracellular matrix of a muscle biopsy taken from a patient with VWA1 disease.^15^ Therefore, the potential role of C-reactive protein in this condition was discussed. Five patients in our cohort received C-reactive protein measurements, but none showed abnormal plasma levels. Given that C-reactive protein is a non-specific marker of inflammation, its significance in VWA1-related disease requires further investigation. Currently, no plasma biomarkers are known to be associated with the condition, and genotypic–phenotypic correlations remain to be established as well. Interestingly, even patients with the most common mutation exhibited variable phenotypic presentations, even within the same family.

The most common associated allele remains the previously reported European founder mutation c.62_71dup, although it was also found in an Iranian and African family (Family 5 and Family 14, respectively). This variant involves an expansion of a 10 bp repeat in Exon 1 of *VWA1*, leading to three copies instead of two compared with normal alleles. This founder mutation was found in the homozygous state in 6 individuals and in the heterozygous state with another likely pathogenic variant in 10 cases. Of the 13 additional variants identified in this cohort, 11 have not been described previously.

Alone in the UK, >26 000 individuals suffer from a form of hereditary neuropathy. With an estimated allele frequency of the most common variant c.62_71dup at 1/847 in the European population, up to 100 individuals may be homozygous or compound heterozygous for the *VWA1* founder mutation in the UK. In Western Europe, North America and Australia, the numbers are even higher. Therefore, *VWA1* should be included in multiple gene panels covering hereditary neuropathies, muscle dystrophies, hereditary spastic paraplegia and SMA to reduce diagnostic delay and identify a large number of undiagnosed individuals.

Ongoing phenotyping of individuals with VWA1 disease will further facilitate the clinical characterization of this newly described condition, while genetic and functional studies remain necessary to understand the link between nerve and muscle pathology observed in an increasing number of neuromuscular disorders.

## Supplementary Material

fcae377_Supplementary_Data

## Data Availability

Anonymized data from participants will be available on request. Data relating to Family 7 are held in the National Genomic Research Library (https://doi.org/10.6084/m9.figshare.4530893.v7). Details of how to access these data are available at www.genomicsengland.co.uk/research/academic/join-gecip.
