# Supplementary material for: Autosomal recessive VWA1-related disorder: comprehensive analysis of phenotypic variability and genetic mutations
Source: Brain Commun. 2024 Oct 28;6(6):fcae377. doi: 10.1093/braincomms/fcae377 (PMC11535570; doi:10.1093/braincomms/fcae377)
Supplement: fcae377_Supplementary_Data [file fcae377_supplementary_data.zip › Supplementary material.pdf]

Supplementary Table 1: Clinical and genetic findings in individuals with VWA1-disease.

a) Scapular winging, dysmorphic signs (dolichocephaly, frontal bossing, high arched palate, bilateral 5th finger clinodactyly, elevated fingertip pads, accessory forked crease on right, creases bilaterally, partial 2,3 syndactyly in feet);
b) Scapular winging, hypermobility and hyperlaxity of fingers, wrists, elbows, toes and feet;
c) Tongue fasciculations, when tired articulation/speech difficulties.

Abbreviations: M=male; F=female; y=years; m=months; NA=not applicable; N=feature not present; Y=feature present; UK=unknown.

		Family1 Patient 1	Family 2 Patient 2	Family 3 Patient 3	Family 4 Patient 4	Family 5 Patient 5
Demographics	**Current age**	27y	19y	6y	14y	13y
	**Age of onset**	1y	17m	2.5y	16m	12y
	**Sex**	UK	M	M	M	M
	**Ethnicity**	Moroccan Jewish	European	European	Mexican	Iranian
	**Consanguinity**	UK	N	N	UK	Y
Early disease stage	**Initial presentation**	Gait disturbances Foot deformity	Gait disturbances Foot deformity Delayed walking	Gait disturbances Foot deformity	UK	Difficulty in sitting and standing up, climbing stairs, muscle stiffness
	**Frequent falls**	Y	Y	Y	Y	Y
	**Contractures**	Ankle	Ankle	Ankle	Ankle	UK
	**Myalgia**	N	N	Y	Y	UK
	**Progression pattern**	Distal LL	Distal LL -> Distal UL -> Proximal LL & UL	Distal LL-> Proximal LL	Distal LL	Distal LL & Proximal LL
	**Age of UL involvement**	NA	2nd decade	NA	NA	NA
	**Progression rate**	Slow	Slow	Slow	Slow	Slow
Clinical symptoms and signs	**Muscle weakness**	Distal LL	Distal LL > Distal UL > Proximal UL, LL	Distal LL > Proximal LL	Distal LL	Distal LL > Proximal LL
	**Foot drop**	Y	Y	Y	Y	UK
	**Muscle atrophy**	N	N	Extremities	Distal LL	UK
	**Muscle hypotonia**	N	N	Y (UL)	N	Y
	**Spasticity**	N	N	Y (LL)	UK	Y
	**Hyporeflexia**	N	Y	Y	UK	UK
	**Plantars**	Upgoing	Downgoing	Upgoing	UK	UK
	**Sensory deficits**	N	N	N	UK	UK
	**Foot deformities**	Pes planus Talipes equinovarus	Pes planus Talipes equinovarus	Pes planus Talipes equinovarus	Pes planus	N
	**Skeletal deformities**	N	N	N	Scoliosis	N
	**Other**	NA	NA	a)	NA	NA
Diagnostic tests	**CK (U/L)**	ND	292	838	1047	2410
	**NCS**	Axonal motor neuropathy	Axonal motor neuropathy	Axonal motor neuropathy	Axonal motor neuropathy	ND
	**EMG**	Chronic denervation UL & LL Acute denervation distal LL	Chronic denervation	Chronic denervation LL	Chronic denervation LL	Chronic myogenic changes LL
	**Muscle biopsy**	ND	ND	ND	ND	Dystrophic pattern
Genetic testing		Compound heterozygous c.455_456del, p.Gly152AlafsTer45 c.548C>G, p.Ser183Ter	Compound heterozygous c.62_71dup, p.Gly25ArgfsTer74 c.1014_1017dup, p.Ile340ArgfsTer106	Compound heterozygous c.62_71dup, p.Gly25ArgfsTer74 c.1169_1217del, p.Leu390ProfsTer133	Compound heterozygous c.62_71dup, p.Gly25ArgfsTer74 c.277T>C, p.Ser93Pro	Homozygous c.62_71dup, p.Gly25ArgfsTer74

		Family 6<br>Patient 6	Family 7<br>Patient 7	Family 8<br>Patient 8	Family 9<br>Patient 9	Family 10<br>Patient 10
Demographics	Current age	3y	74y	6y	4y	19y
	Age of onset	2.5y	2y	18m	1y	8m
	Sex	M	F	M	M	M
	Ethnicity	European	European	European	European<br>Arab	European<br>Pakistani
	Consanguinity	N	N	N	N	Y
Early disease stage	Initial presentation	Gait disturbances Foot deformity	UK	Gait disturbances<br>Foot deformity	Foot deformity	Delayed crawling and walking,<br>Foot deformity
	Frequent falls	Y	Y	Y	N	Y
	Contractures	Ankle	N	Ankle, knee, hip	Ankle	N
	Myalgia	N	N	Y	N	Y
	Progression pattern	Distal LL -><br>Axial	Distal LL -><br>Proximal LL	Distal LL & Proximal LL<br>& Axial	Distal LL -><br>Neck flexor	Distal LL -><br>Distal UL
	Age of UL involvement	NA	NA	4y	NA	6y
	Progression rate	Slow	Slow	Slow	Slow	Slow
Clinical symptoms and signs	Muscle weakness	Distal LL &<br>Trunk flexors	Distal LL > Proximal LL	Distal LL > Proximal LL<br>> Distal UL	Distal LL &<br>Neck flexor	Distal LL ><br>Distal UL
	Foot drop	Y	Y	Y	UK	Y
	Muscle atrophy	Distal LL	Distal LL	Proximal and distal LL,<br>Serratus anterior	Distal LL	Distal LL> distal UL
	Muscle hypotonia	N	Y	N	N	N
	Spasticity	N	N	N	N	UK
	Hyporeflexia	Y	Y	Y	Y	Y
	Plantars	Downgoing	Downgoing	Downgoing	Downgoing	Downgoing
	Sensory deficits	N	Light touch and<br>pinprick reduced<br>distal LL	Light tough and<br>vibration sensation<br>reduced distal LL	UK	N
	Foot deformities	Pes cavus	Pes cavus	Pes planus	Pes cavus<br>Talipes equinovarus	Talipes equinovarus
	Skeletal deformities	N	N	N	N	N
	Other	N	N	b)	N	N
Diagnostic tests	CK (U/L)	374	ND	126	Normal	ND
	NCS	Axonal motor<br>neuropathy	Axonal motor<br>neuropathy	Axonal motor<br>neuropathy	Axonal motor<br>neuropathy	Axonal motor<br>neuropathy
	EMG	Chronic denervation<br>distal LL	Acute and chronic<br>denervation distal LL	Chronic denervation LL	ND	Chronic denervation LL
	Muscle biopsy	ND	ND	Neurogenic pattern	ND	ND
Genetic testing		Compound<br>heterozygous<br>c.62_71dup,<br>p.Gly25ArgfsTer74<br>c.1088_1091dup,<br>p.Tyr364Ter	Homozygous<br>c.62_71dup,<br>p.Gly25ArgfsTer74	Compound<br>heterozygous<br>c.62_71dup,<br>p.Gly25ArgfsTer74<br>c.949del,<br>p.Ala317LeufsTer34	Compound<br>heterozygous<br>c.62_71dup,<br>p.Gly25ArgfsTer74<br>c.212T>C, p.Leu71Pro	Homozygous<br>c.763del,<br>p.Ala255LeufsTer29

		Family 11 Patients 11	Family 11 Patient 12	Family 12 Patient 13	Family 12 Patient 14
Demographics	**Current age**	31y	33y	10y	8y
	**Age of onset**	2y	1y	2y	4y
	**Sex**	M	M	M	M
	**Ethnicity**	European		European	
	**Consanguinity**	N		N	
Early disease stage	**Initial presentation**	Standing and gait disturbances Foot deformity		Gait disturbances	
	**Frequent falls**	Y	UK	Y	
	**Contractures**	Y		Ankle	N
	**Myalgia**	N		Y	
	**Progression pattern**	Distal LL -> Distal UL	Distal LL -> Distal UL -> Proximal LL & UL	Distal LL	Distal LL -> Proximal LL
	**Age of UL involvement**	28y	16y	NA	
	**Progression rate**	Slow		Slow	
Clinical symptoms and signs	**Muscle weakness**	Distal LL > Distal UL	Distal LL& Distal UL > Proximal LL.> Proximal UL	Distal LL	Distal LL > Proximal LL
	**Foot drop**	Y	UK	Y	
	**Muscle atrophy**	Distal LL> distal UL		N	
	**Muscle hypotonia**	N		N	
	**Spasticity**	N		N	
	**Hyporeflexia**	N	Y	N	
	**Plantars**	Downgoing		Downgoing	
	**Sensory deficits**	N	Joint position sense altered LL	N	
	**Foot deformities**	Pes cavus Talipes equinovarus		Pes cavus Talipes equinovarus	Pes planus Talipes equinovarus
	**Skeletal deformities**	N	Scoliosis	Pectus excavatum	
	**Other**	c)		N	
Diagnostic tests	**CK (U/L)**	1530	998	ND	
	**NCS**	Axonal motor neuropathy with mild sensory neuropathy		ND	
	**EMG**	Chronic denervation LL & UL		ND	
	**Muscle biopsy**	ND	Compatible with neurogenic process	ND	
Genetic testing		Compound heterozygous c.473T>C, p.Leu158Pro c.879del, p.Arg293SerfsTer58		Homozygous c.62_71dup, p.Gly25ArgfsTer74	

		Family 13 Patient 15	Family 13 Patient 16	Family 14 Patient 17	Family 14 Patient 18
Demographics	**Current age**	11y	6y	7y	2y
	**Age of onset**	3y	3y	2y	2y
	**Sex**	F	F	M	F
	**Ethnicity**	European		Black Zambian	
	**Consanguinity**	N		N	
Early disease stage	**Initial presentation**	Gait disturbances	Foot deformity	Foot deformity	
	**Frequent falls**	N		N	
	**Contractures**	Ankle		Ankle	
	**Myalgia**	Y	N	N	
	**Progression pattern**	Distal LL -> Distal UL		Distal LL	
	**Age of UL involvement**	UK		NA	
	**Progression rate**	Slow		Slow	
Clinical symptoms and signs	**Muscle weakness**	Distal LL > Distal UL		Distal LL	
	**Foot drop**	Y (only on the left)	Y	N	
	**Muscle atrophy**	Y		N	
	**Muscle hypotonia**	N		N	
	**Spasticity**	N		N	
	**Hyporeflexia**	N		UK	
	**Plantars**	Downgoing		UK	
	**Sensory deficits**	N		UK	
	**Foot deformities**	Pes cavus and talipes equinovarus on the left; Pes planus on the right	Bilateral Pes cavus	Pes planus	
	**Skeletal deformities**	N		N	
	**Other**	N		N	
Diagnostic tests	**CK (U/L)**	126	ND	ND	
	**NCS**	Axonal motor neuropathy		Axonal motor and sensory neuropathy	ND
	**EMG**	Chronic denervation LL		Normal	ND
	**Muscle biopsy**	ND		ND	
Genetic testing		Homozygous c.62_71dup, p.Gly25ArgfsTer74		Compound heterozygous c.62_71dup, p.Gly25ArgfsTer74 c.662dup, p. Glu222GlyfsTer65	

		Family 15 Patient 19	Patient 20
Demographics	Current age	9y	11y
	Age of onset	Congenital	
	Sex	M	F
	Ethnicity	European	
	Consanguinity	N	
Early disease stage	Initial presentation	Foot deformity	
	Frequent falls	Y	N
	Contractures	Ankle, Popliteal	N
	Myalgia	Y	
	Progression pattern	NA	
	Age of UL involvement	NA	
	Progression rate	Stable disease	
Clinical symptoms and signs	Muscle weakness	N	Proximal LL
	Foot drop	N	
	Muscle atrophy	N	
	Muscle hypotonia	N	
	Spasticity	N	
	Hyporeflexia	N	
	Plantars	Downgoing	
	Sensory deficits	N	
	Foot deformities	Pes cavus<br>Talipes equinovarus	
	Skeletal deformities	N	
	Other	Dystonia LL	N
Diagnostic tests	CK (U/L)	Normal	ND
	NCS	ND	
	EMG	ND	
	Muscle biopsy	ND	
Genetic testing		Compound heterozygous<br>c.62_71dup, p.Gly25ArgfsTer74<br>c514C>T p.Arg172Ter	

Supplementary Table 2. **Frequency of the most common *VWA1* variant across databases.**

**cDNA/Coding Sequence Change [NM_022834.5]**	c.62_71dup
**Protein Change [NP_001001683.1]**	p.Glu25ArgfsTer74
**Exon or intron (number position) [GrCh38/hg38]**	exon 1 of 3 before position 131 of 132 (splicing, coding, NMD)
**Codon change**	GGCGCGGAGC (10bp)
**Variant type**	Frameshift
**DbSNP ID**	rs749383814
**GnomAD v4.0.0**	ƒ = 0.0009314 (1, 1120; European Non-Finnish 0.001053)
**GnomAD v3.1.2**	ƒ = 0.0006689 (0, 100; European Non-Finnish 0.0009556)
**GnomAD v2.1.1**	ƒ = 0
**NCBI alfa**	ƒ = 0.00007 (0, 2/28256)
**TopMed**	ƒ = 0.0000477
**Regeneron Genetics Center (RGC)**	ƒ = 0.0000328 (SAS 0.0000566)
**UK Biobank**	ƒ = 0.00118 (0, 862)
**ExAC v1.0**	Not found
**Iranome**	n/a
**GME Variome**	n/a
**54KJPN**	ƒ = 0.000028
**38KJPN**	ƒ = 0.000052
**14KJPN**	ƒ =0.000071
**GenomeAsia**	Not found
**ESP 6500**	n/a
**KAVIAR**	ƒ = 0.0013
**KOVA**	Not found
**NyuWa/ NCVD**	ƒ =0.00017053
**Middle East and North Africa (MENA)**	Not found
**IndiGenomes**	Not found
**South Asian Genomes & Exomes (SAGE)**	Not found
**Geno2MP**	Not found
**Human Genetic Variation Database**	Not found
**ABraOM (Brazilian genomic variants)**	Not found
**In-house Database**	ƒ =0.0002 (0, 6)

Supplementary Table 3: Additional information for all *VWA1* variants identified in the present cohort.

Annotations generated using VEP (www.ensembl.org/Tools/VEP).

*Single submission from GeneDx likely corresponds to Family 2 described here.

Family	#Uploaded variant	Location (GRCh38)	Allele	Consequence	IMPACT	EXON	HGVSc	HGVSp	Existing variation	ClinVar (20th Oct 2023)	SIFT	PolyPhen	gnomADe_AF	gnomADg_AF	CADD_PHRED	SpliceAI_pred_DP_AG	SpliceAI_pred_DP_AL	SpliceAI_pred_DP_DG	SpliceAI_pred_DP_DL	SpliceAI_pred_DS_AG	SpliceAI_pred_DS_AL	SpliceAI_pred_DS_DG	SpliceAI_pred_DS_DL
1	NM_022834.5:c.455_456del	1:1437307-1437309	-	frameshift_variant	HIGH	2/3	NM_022834.5:c.455_456del	NP_073745.2:p.G152Afs*45	-	-	-	-	-	-	-	15	-23	-35	47	0.32	0	0	0
1	NM_022834.5:c.548C>G	1:1437401-1437401	G	stop_gained	HIGH	2/3	NM_022834.5:c.548C>G	NP_073745.2:p.S183*	rs750227698	VCV001377417.2 (P)	-	-	-	-	36	-41	3	28	-47	0	0	0	0
2	NM_022834.5:c.1014_1017dup	1:1439466-1439466	CGTC	frameshift_variant	HIGH	3/3	NM_022834.5:c.1014_1017dup	NP_073745.2:p.I340Rfs*106	-	VCV001318173.3 (LP*)	-	-	-	-	-	-	-	-	-	-	-	-	-
3	NM_022834.5:c.1169_1217del	1:1439617-1439666	-	frameshift_variant	HIGH	3/3	NM_022834.5:c.1169_1217del	NP_073745.2:p.L390Pfs*133	-	-	-	-	-	-	-	-	-	-	-	-	-	-	-
4	NM_022834.5:c.277T>C	1:1437130-1437130	C	missense_variant	MODERATE	2/3	NM_022834.5:c.277T>C	NP_073745.2:p.S93P	-	-	deleterious(0)	probably_damaging(0.973)	-	-	24.9	42	-1	23	3	0	0	0.01	0
6	NM_022834.5:c.1088_1091dup	1:1439540-1439540	GCTA	stop_gained,frameshift_variant	HIGH	3/3	NM_022834.5:c.1088_1091dup	NP_073745.2:p.Tyr364*	-	VCV001675331.10 (LP)	-	-	-	-	-	-	-	-	-	-	-	-	-
8	NM_022834.5:c.949del	1:1439397-1439398	-	frameshift_variant	HIGH	3/3	NM_022834.5:c.949del	NP_073745.2:p.A317Lfs*34	-	-	-	-	-	-	-	44	22	-33	-38	0	0	0	0
9	NM_022834.5:c.212T>C	1:1437065-1437065	C	missense_variant	MODERATE	2/3	NM_022834.5:c.212T>C	NP_073745.2:p.L71P	rs747475277	VCV001695698.2 (VUS)	deleterious(0)	possibly_damaging(0.748)	4.06E-05	4.60E-05	27.6	-34	49	8	13	0	0	0	0
10	NM_022834.5:c.763del	1:1439211-1439212	-	frameshift_variant	HIGH	3/3	NM_022834.5:c.763del	NP_073745.2:p.A255Lfs*29	-	-	-	-	-	-	-	-46	26	25	-21	0	0	0	0
11	NM_022834.5:c.473T>C	1-1437326-1437326	C	missense_variant	MODERATE	2/3	NM_022834.5:c.473T>C	NP_073745.2: p.Leu158Pro	rs1638567522	-	deleterious(0)	probably_damaging(0.993)	-	-	26.9	-4	34	0	-2	0.02	0	0	0
11	NM_022834.5:c.879del	1:1439327-1439328	-	frameshift_variant	HIGH	3/3	NM_022834.5:c.879del	NP_073745.2:p.Arg293Serfs*58	rs746212067	VCV000830328.5	-	-	4.829e-05	6.579e-05	-	8	16	16	32	0	0	0.02	0
14	NM_022834.5:c.662dup	1:1439111-1439111	-	frameshift_variant	HIGH	3/3	NM_022834.5:c.662dup	NP_073745.2:p.Glu222GlyfsTer65	-	-	-	-	-	-	-	-30	-13	-10	-33	0	0	0	0
15	NM_022834.5:c.514C>T	1:1437367-1437367	T	stop_gained	HIGH	2/3	NM_022834.5:c.514C>T	NP_073745.2:p.Arg172Ter	rs749945726	-	-	-	4.01e-06	1.971e-05	22.1	25	-45	3	-43	0	0	0	0
2-9, 12-15	NM_022834.5:c.62_71dup	1:1435819-1435819	GCGCGGAGCG	frameshift_variant,splice_region_variant	HIGH	1/3	NM_022834.5:c.62_71dup	NP_073745.2:p.G25Rfs*74	-	VCV000830327.8 (P)	-	-	-	-	-	-	-	-	-	-	-	-	-
